# Adrenal Metastasis of Hepatocellular Carcinoma in Patients following Liver Resection or Liver Transplantation: Experience from a Tertiary Referral Center

**DOI:** 10.1155/2018/4195076

**Published:** 2018-07-29

**Authors:** Eva M. Teegen, Martina T. Mogl, Johann Pratschke, Nada Rayes

**Affiliations:** ^1^Department of Surgery, Charité-Universitätsmedizin Berlin, Augustenburgerplatz 1, 13353 Berlin, Germany; ^2^Department of Visceral, Transplant, Thoracic and Vascular Surgery, University Hospital Leipzig, Liebigstr. 20, 04103 Leipzig, Germany

## Abstract

**Introduction:**

Adrenal metastasis of hepatocellular carcinoma (HCC) is a rare entity and can be treated by resection, local ablative therapy, or systemic therapy. Unfortunately, data about treatment outcome, especially in liver transplant recipients, are rare.

**Patients and Methods:**

From 2005 to 2015, 990 liver resections and 303 liver transplantations because of HCC were performed at our clinic. We retrospectively analyzed treatment outcome of the patients with metachronous adrenal metastasis of HCC, who received either resection, local ablation, or surveillance only.

**Results:**

10 patients were identified (0.8%). 7 patients received liver transplantation for primary HCC therapy, 3 liver resection, and 1 a local ablative therapy. 8 patients underwent adrenalectomy (one via retroperitoneoscopy), one was treated with local ablation, and one had surveillance only. Seven out of eight patients had no surgical complications and one experienced a pancreatic fistula, treated conservatively. 37.5% of the resected patients had recurrence 1 year after adrenalectomy and 75% after 2 years. The mean survival time after primary diagnosis of HCC was 96.6±22.4 months. After adrenalectomy, the mean survival time was 112.4±25.2 months. The mean time until tumor recurrence was 13.2±3.8 in the total cohort and 15.8±3.8 months in patients after adrenalectomy. The estimated overall survival after adrenalectomy was 77.2±17.4 months.

**Conclusion:**

Metachronous adrenal metastasis occured in less than 1% of HCC patients. Adrenalectomy is a safe procedure and leads to acceptable survival rates even after liver transplantion. Therefore, it should be performed whenever the primary tumor is well controlled and the patient is in adequate physical condition.

## 1. Introduction

Early detection using MRI and new treatment options like laparoscopic surgery and local ablative therapy continuously improved survival outcomes of hepatocellular carcinoma (HCC) during the last decades. Therapeutic modalities for the primarius range from surgical resection, systemic therapy, and local ablative methods to liver transplantation. However, HCC is still the second leading cause of cancer death according to the worldwide statistical report of the World Health Organization from 2012. Extrahepatic metastases of HCC usually appear simultaneously with the primary tumor but they can also occur during the further clinical course with or without additional tumor recurrence [1]. Particularly patients with advanced intrahepatic lesions (tumor stages T3 and T4), vascular invasion, elevated tumor markers, and viral hepatitis have a higher risk for extrahepatic metastases from HCC with an incidence of 13.5%-42%. Furthermore, extrahepatic metastases especially after liver transplantation are more aggressive compared to those occurring in patients without liver transplantation [2–6]. Generally, HCC disseminates via the lymphatic or vascular pathway affecting lymph nodes (33.8%) and most frequently the lung (53.8-74.5%), followed by the bones (24.8-38.5%) and the adrenal glands (8-19.1%) [1, 2, 4, 6–9]. Different therapeutic strategies like local ablative methods and systemic therapy as well as surgery have been considered for the therapy of adrenal metastasis of HCC. Local treatment including transarterial chemoembolization (TACE), percutaneous ethanol injection (PEI), radiotherapy, and radiofrequency ablation provides survival rates of 42–42.5% at 1 year and a median survival of 11.1–13.6 months [9–13]. Patients with isolated extrahepatic metastasis and preserved liver function may generally benefit from a surgical resection [1, 7].

Previous reports of treatment outcome especially following liver transplantation are rare and mainly published from Asian countries. Due to the small number of patients, prospective studies comparing treatment outcome have not been performed. Therefore, we analyzed our data from a Western high volume liver center to evaluate incidence, outcome, and survival of patients with adrenal metastasis of HCC after liver resection and liver transplantation.

## 2. Patients and Methods

From January 2006 to December 2015 990 liver resections and 303 liver transplantations for HCC were performed at our clinic. We detected ten patients with metachronous adrenal metastases. Eight patients underwent adrenalectomy and one patient was treated with local ablative therapy whereas one adrenal metastasis remained untreated due to the patient's physical condition and tumor progression. Data was retrospectively extracted from our digital patient data base. Diagnosis of adrenal metastasis was made by either computed tomography or magnetic resonance imaging. Patients were either grouped to those with or without liver transplantation or those with or without adrenalectomy to analyze survival and tumor recurrence seperately. The primary endpoint of this study was survival after adrenalectomy; secondary endpoints were the overall survival after primary diagnosis of HCC and the time until tumor recurrence. Statistical analysis was done using EXCEL (Version 15.21.1, 2016 Microsoft) with the add-in XLSTAT (Version 2016.02.27941, Addinsoft, New York, USA). The analysis was explorative. Kaplan-Meier-analysis was used for survival rates.

## 3. Results

**Figure 1:**
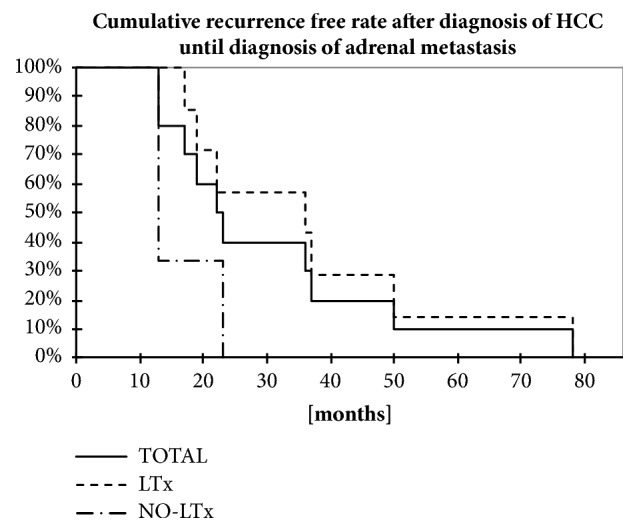
Display shows Kaplan-Meyer-Analysis of recurrence-free rate after the primary diagnosis of HCC until the occurring diagnosis of an adrenal metastasis depending on the primary treatment of the HCC either liver transplantation or resection/afterloading. N=10.

The demographic data are shown in Table 1. In eight patients, an adrenalectomy was performed, one patient with bilateral adrenal metastasis was treated with afterloading, and one metastasis remained untreated due to severe tumor progress and reduced physical condition of the patient. There were seven male (70.0%) and three female patients (30.0%) in the total cohort and six male (75.0%) and two female (25.0%) patients in the adrenalectomy group (Table 1). The mean age at the time of HCC diagnosis was 60.8±6.5 years (versus 60.9±6.5 years in the adrenalectomy group) and the mean age at the time of diagnosis of the adrenal metastasis was 63.9±7.1 years (versus 64.4±7.1 years in the adrenalectomy group). The mean interval between detection of primary tumor and adrenal metastasis was 30.8±6.5 months in the total cohort, 37.0±8.2 months in patients after liver transplantation (versus 16.3±3.3 in patients without liver transplantation), and 34.0±7.7 months in the adrenalectomy group (versus 18.0±5 in nonadernalectomy group) (Table 1 and Figure 1). 50% of the patients showed BCLC stage B, three stage A, and one C at the time of HCC diagnosis. 50% suffered from a multifocal HCC with more than 3 tumor nodes; three patients presented 1 and one patient 3 HCC nodes. The average HCC tumor size was 42.0±23.0 in the total cohort. The primary HCC was resected in three patients (30%) and seven patients underwent liver transplantation (70%). The only patient in whom the HCC in the liver was treated with local ablative therapy developed bilateral adrenal metastasis later on which were again treated locally (Table 1). Metastases were located on the left side (n=5), on the right side (n=4), or on both sides (n=1) (Table 1). The mean size of the adrenal metastasis was 45.6+±21.4 mm (versus 45.1±21.6 mm in the adrenalectomy group, Table 1). Seven patients were operated via an open approach and only one patient underwent a retroperitoneoscopic procedure (Table 1). Seven out of eight patients had no surgical complications after adrenalectomy; one experienced a pancreatic fistula, which was treated conservatively (Table 1).

The tumor recurrence rate one year after treatment of the adrenal metastasis was 37.5% in the adrenalectomy group (n=8, Table 1). After two years, six out of eight patients (75%) after adrenalectomy showed tumor recurrence at the following sites: intrahepatic recurrence (n=4), bone metastases (n=3), brain metastasis (n=1), adrenal metastasis on the contralateral side (n=2), and pulmonary recurrence (n=2) after 28 months (Table 1). Only one patient developed metastases at two extrehepatic sites and one patient an intrehepatic and extrehepatic recurrence. The patient with local ablative therapy for his adrenal metastasis experienced tumor recurrence nine months after therapy (Table 1).

**Figure 2:**
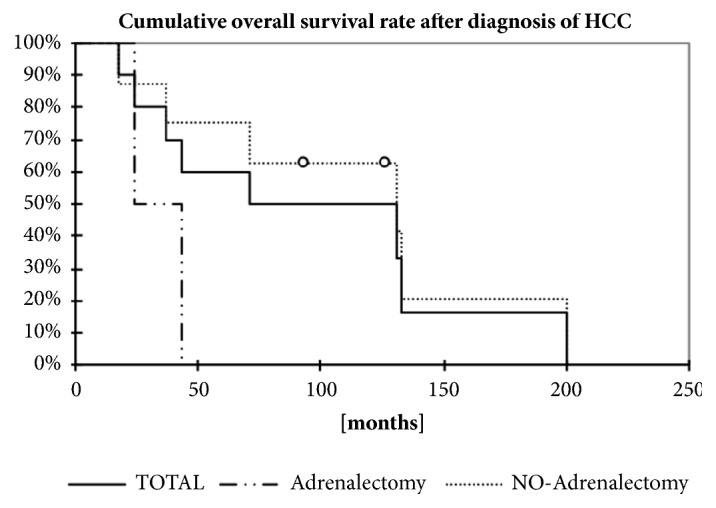
Display shows Kaplan-Meyer-Analysis of estimated overall survival after the diagnosis of adrenal metastasis depending on the treatment of the adrenal metastasis. N=10.

The median time until tumor recurrence (tumor progression free survival) was 12.5 (0-28) in the total cohort, 19 (4-28) months in patients after adrenalectomy, and 4.5 (0-9) months in no-adernalectomy group (Figure 2 and Table 1). The median survival time after primary diagnosis of HCC was 82 (18-200) months (Figure 2 and Table 1). In patients after adrenalectomy, the median survival time after primary diagnosis of HCC was 109.5 (17.5-200) months versu 31.5 (18-45) in the group without adrenalectomy (Figure 2 and Table 1). The median survival after adrenalectomy was 69 (0.5-122) months and 89 (0.5-122) months after adrenalectomy following liver transplantation (Table 1).

## 4. Discussion

The adrenal gland is a common site of metastatic HCC, but still remaining a rare entity in clinical routine [14]. We identified six patients after liver transplantation, three after liver resection, and one after local therapy, who developed adrenal metastasis at a mean of 30.8±19.4 months after diagnosis of primary HCC. The timepoint of metastatic development may depend on the initial treatment for HCC. In previous series, adrenal metastasis occurs significantly later after liver transplantation than after liver resection, a fact that we could confirm in our patients [15]. One reason might be that patients who were treated by liver transplantation had a smaller primarius as they had to fullfill the Milan criteria [16]. In contrary, most of our patients showed higher BCLC stages and were transplanted beyond Milan criteria following living donation. Furthermore, these patients were on immunosuppression, which might facilitate the development of malignancies. Previous investigations showed a risk for HCC recurrence after liver transplantation of 25.0% with most of the patients presenting extrahepatic recurrence (80.0%). Particularly patients with a primary HCC beyond Milan criteria or with macrovascular tumor invasion were significantly associated with extrahepatic recurrence. But among patients with primary HCC within the Milan criteria, extrahepatic tumor recurrence was only associated in cases of higher DNA-Index (DNA-index>1.5, p=0.013) [17]. In case of HCC recurrence, metastases should be treated in the right way, to improve patients' survival.

Adrenalectomy was already performed for recurrence of HCC in different settings after liver transplantation and extensive multivisceral resections or for palliative metastasectomy [18–20]. Minimal-invasive procedures were established for small tumor size and certain risk factors and belong to the standard today [21–25]. Most of our patients received a primarily open surgical procedure, because of malignancy, a larger size, and prior surgery with the risk for abdominal adhesion. But we also owed a certain lack of experience with those patients during the former time. In nowadays, due to further development and experience with laparoscopic adrenalectomies in benign dignities, it should be performed whenever possible and retroperitoneoscopic adrenalectomy may serve as a good alternative in case of severe adhesion [26]. Alternatively to metastasectomy, a variation of nonsurgical treatment options like PEI, TACE, radiation, and radiofrequency ablation were also reported and recent chemotherapeutic protocols achieved palliative control of adrenal metastasis [27–31].

The first analysis and comparison of the different therapeutic modalities were published in France in 1998, including 13 patients [32]. The authors reported a longer survival in the patients with surgical resection compared to those with PEI and radiation, but two out of seven patients died during the postoperative course. Therefore, the investigators recommended surgical treatment for only selected patients [33]. Other studies showed that adrenalectomy after curative treatment for primary HCC could definitely improve survival rates (68% at 1 year) in comparison to nonsurgical treatments (42–42.5% at 1 year) [10–13, 33, 34]. Furthermore, depending on the curative HCC treatment, Ha et al. could show especially longer recurrence-free and survival rates for patients who initially received a liver transplantation compared to those after curative liver resection [1]. According to that, our patients after liver transplantation with adrenal recurrence of a HCC also present a longer recurrence-free period after treatment of the primarius and longer overall survival rates after adrenalectomy compared to the group whose HCC was primarily resected with no severe surgical complications. Therefore, the aggressive treatment of HCC metastases in the adrenal gland by adrenalectomy can improve long term survival despite the risk for recurrence and perioperative challenges after prior major abdominal surgery.

## 5. Conclusion

Limitations of the present study are the small number of patients and the retrospective design. However, our data are consistent with the data from the few existing former studies. Adrenalectomy is a safe treatment for adrenal metastases of HCC even after liver transplantation and should be performed whenever the primary tumor is well controlled and when the patient is in adequate physical condition [10, 15, 35, 36].

## Figures and Tables

**Table 1:** Demographic data and survival dates: N=10 (BCLC= Barcelona clinic liver cancer; HCC= hepatocellular carcinoma; SD= standard deviation).

	Total cohort (n=10)	Adrenalectomy (n=8)	Afterloading (n=1)	No Therapy (n=1)
Gender				

male	7 (70.0%)	6 (75.0%)	1 (100%)	0
female	3 (30.0%)	2 (25.0%)	0	1 (100.0%)

Age at time of HCC (SD) [years]	60.8 (6.5)	60.9 (6.5)	54	67

Age at time of adrenal metastasis (SD) [years]	63.4 (7.1)	64.4 (7.1)	55	69

Interval since HCC (SD) [months]	30.8 (6.5)	34.0 (7.7)	13	23
		37.0 (8.2) LTx		

BCLC stage at time of diagnosis of HCC				

A	3 (30.0%)	2 (25.0%)	1 (100%)	
B	5 (50.0%)	5 (62.5%)		
C	2 (20.0%)	1 (12.5%)		1 (100%)

Amount of HCC nodes at time of diagnosis				

1	4 (40.0%)	2 (25.0%)	1 (100%)	1 (100%)
3	1 (10.0%)	1 (12.5%)		
multifocal	5 (50.0%)	5 (62.5%)		

Size of largest HCC node (SD) [mm]	42.0 (23.0)	47.0 (20.1)	7	10

Primary therapy of HCC				

Liver resection	2 (30.0%)	1 (12.5%)	0	1 (100%)
Liver transplantation	7 (70.0%)	7 (87.5%)	0	0
Local ablative therapy	1 (10.0%)	0	1 (100%)	0

Side of adrenal metastasis				

right	4 (30.0%)	3 (37.5%)	0	1 (100%)
left	5 (60.0%)	5 (62.5%)	0	0
bilateral	1 (10.0%)	0	1 (100%)	0

Size of adrenal metastasis (SD) [mm]	45.6 (21.4)	45.1 (21.6)	68	27

Type of adrenalectomy				

open	7 (70.0%)	7 (87.5%)	-	-
retroperitoneoscopic	1 (10.0%)	1 (12.5%)	-	-

Surgical complications after adrenalectomy		1 (12.5%)		

Recurrence rate after adrenal metastasis				

After 1 year	-	5 (37.5%)	1 (100%)	-
After 2 years	-	6 (75.0%)	-	-

Type of recurrence				

Hepatic	6 (60.0%)	4 (50.0%)	1 (100%)	1 (100%)
Bone	3 (30.0%)	3 (37.5%)	0	0
Pulmonary	2 (20.0%)	2 (25.0%)	0	0
Brain	3 (30.0%)	1 (12.5%)	0	0
Adrenal contralateral	2 (20.0%)	2 (25.0%)	0	0

Time to progress [months]	12.5 (0-28)	19 (4-28) 20 (4-28) LTx	0	9

Survival after HCC [months]	82 (17.5-200)	109.5 (17.5-200)	43	24
		126 (17.5-200)LTx		

Survival after adrenalectomy [months]	-	69 (0.5-122)	-	-
		81 (0.5-122) LTx		

## Data Availability

The data used to support the findings of this study are available from the corresponding author upon request.
